# Effects of Nitrogen Content on the Structure and Mechanical Properties of (Al_0.5_CrFeNiTi_0.25_)N_x_ High-Entropy Films by Reactive Sputtering

**DOI:** 10.3390/e20090624

**Published:** 2018-08-21

**Authors:** Yong Zhang, Xue-Hui Yan, Wei-Bing Liao, Kun Zhao

**Affiliations:** 1 State Key Laboratory for Advanced Metals and Materials, University of Science and Technology Beijing, Beijing 100083, China; 2 College of Physics and Energy, Shenzhen University, Shenzhen 518060, China; 3 Department of Physics, School of Science, Hebei University of Science and Technology, Shijiazhuang 050000, China

**Keywords:** high-entropy films, phase structures, hardness, solid-solution, interstitial phase

## Abstract

In this study, (Al_0.5_CrFeNiTi_0.25_)N_x_ high-entropy films are prepared by a reactive direct current (DC) magnetron sputtering at different N_2_ flow rates on silicon wafers. It is found that the structure of (Al_0.5_CrFeNiTi_0.25_)N_x_ high-entropy films is amorphous, with x = 0. It transforms from amorphous to a face-centered-cubic (FCC) structure with the increase of nitrogen content, while the bulk Al_0.5_CrFeNiTi_0.25_ counterpart prepared by casting features a body-centered-cubic (BCC) phase structure. The phase formation can be explained by the atomic size difference (δ). Lacking nitrogen, δ is approximately 6.4% for the five metal elements, which is relatively large and might form a BCC or ordered-BCC structure, while the metallic elements in this alloy system all have a trend to form nitrides like TiN, CrN, AlN, and FeN. Therefore, nitride components are becoming very similar in size and structure and solve each other easily, thus, an FCC (Al-Cr-Fe-Ni-Ti)N solid solution forms. The calculated value of δ is approximately 23% for this multicomponent nitride solid solution. The (Al_0.5_CrFeNiTi_0.25_)N_x_ films achieve a pronounced hardness and a Young’s modulus of 21.45 GPa and 253.8 GPa, respectively, which is obviously much higher than that of the as-cast Al_0.5_CrFeNiTi_0.25_ bulk alloys.

## 1. Introduction

High-entropy films (HEFs) are a brand-new type of alloy film, which has been developed recently based on the design concept of high-entropy alloys (HEAs) [1]. HEFs can be defined as multiple-component films with high-entropy mixing. Generally, the HEAs are composed of multi-principal-elements (at least five elements with five at a % ≤ each element content ≤ 35 at %) [2,3]. HEAs feature higher mixing entropy than traditional alloys and tend to form disorder face-centered cubic (FCC) and/or body-centered cubic (BCC) phase structures rather than ordered intermetallic compounds [4,5,6]. Due to severe lattice distortion and solid solution strengthening, attributable to the multi-components, the HEAs show many excellent mechanical properties, such as high-strength, good ductility, and high-wear and corrosion resistances [1]. Based on a similar scientific concept, HEFs have been designed and investigated gradually and show a great potential for application in the coating industry [7,8,9,10,11,12]. To date, many excellent properties of HEFs have been discovered and studied, such as high-wear resistance [13,14], high-corrosion resistance [15,16,17], diffusion-barriers effects [18,19,20], solar-thermal-conversion effects [10,21], plastic-deformation characteristics [22,23], thermal stabilities [7,24,25], and soft magnetic properties [26]. However, there are few theories that can explain the mechanism of the phase formation of the HEFs.

The phase formation and mechanical properties of (Al_0.5_CrFeNiTi_0.25_)N_x_ high-entropy thin films with different N_2_ flow rates are studied in this paper. The phase structures and mechanical properties of Al_x_CrFeNiTi_0.25_ (x: molar ratio, x = 0, 0.25, 0.5, 0.75, and 1.0) bulks have previously been studied systematically [27]. Herein, the optimal composition of Al_x_CrFeNiTi_0.25_ (x = 0.5) alloy is selected as a magnetron-sputtering target to explore the phase formation mechanism and mechanical properties of high-entropy thin films. Significantly, the phase structures of high-entropy thin films transform from amorphous to FCC with the increase of nitrogen content, while the bulk Al_0.5_CrFeNiTi_0.25_ alloy attains BCC phase structure. Phase formation mechanism is explored from both theoretical calculations and experiments. Concerning high entropy thin films, the ability for solid solution structure formation is first discussed from the atom radius difference (δ). This study can help to provide new insights into understanding the phase formation mechanism of multi-component alloy thin film solids with small atoms such as nitrogen.

## 2. Materials and Methods

**Figure 1:**
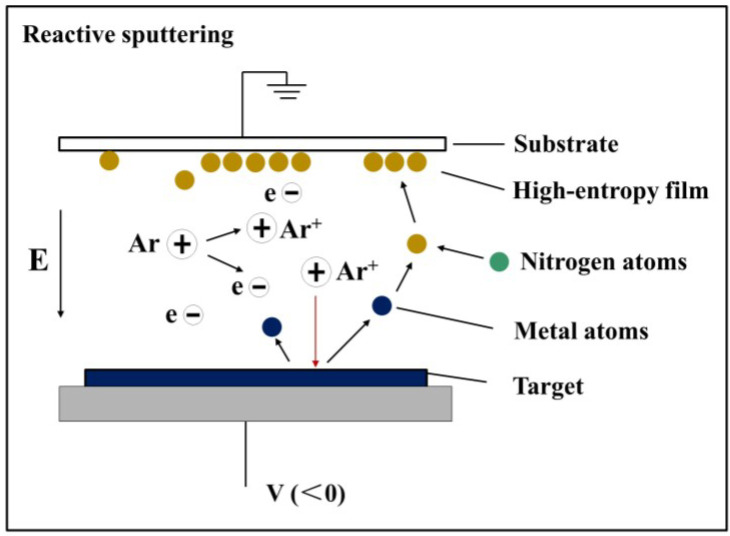
The schematic diagram of reactive sputtering.

The (Al_0.5_CrFeNiTi_0.25_)N_x_ films were deposited on p-type Si (100) wafers by a direct current (DC) magnetron sputtering using non-equal atomic ratio Al_0.5_CrFeNiTi_0.25_ targets of Φ 60 mm in diameter and 2 mm in thickness. The alloy target was prepared by arc-melting and the smelting was repeated at least five times to ensure uniform mixing of components. Prior to deposition, the Si substrates were cleaned sequentially and rinsed by acetone, alcohol, and distilled water in an ultrasonic bath. Pre-sputtering was an effective way to remove oxide or contaminants on the surface of the target. When the base pressure held at 2.0 × 10^−4^ Pa, high purity argon was injected into the vacuum chamber and the target was cleaned by argon ion bombardment for 15 min at a power of 100 W. The deposition of the (Al_0.5_CrFeNiTi_0.25_)N_x_ films were carried out in an Ar + N_2_ mixed atmosphere under a DC power of 100 W with a working pressure of 0.5 Pa. The schematic diagram of reactive sputtering is shown in Figure 1. The metal atoms escaped the surface of the target due to the bombardment of high energy particles. Due to the nitrogen atmosphere, different metal atoms reacted with N-atoms and were deposited on the substrates in the end. During the deposition, the total flow rate of Ar + N_2_ was maintained at 30 standard cubic centimeters per minute (sccm) and the ratio was N_2_/(Ar + N_2_) and R_N2_ was controlled at 0, 10%, 20%, 30%, 40%, and 50%, respectively. The work distance between the substrate and the target was 60 mm and the deposition time was maintained at 60 min. No external heating or bias was used on the substrate during the deposition process.

The crystal structures of (Al_0.5_CrFeNiTi_0.25_)N_x_ films were analyzed by a glancing-incidence (1°) X-ray diffractometer (XRD, BRUKERD8 Discover, Bruker, Karlsruhe, Germany) using the Cu K_α_ radiation at a scanning rate of 4°/min. The scanning step was 0.02° with a scanning range of 20°–90°. The morphology studies and thickness measurements were carried out using field-emission scanning electron microscopy (SEM, Auriga Field Emission Scanning Electron Microscope, Carl Zeiss, Jena, Germany) equipped with an Energy Dispersive X-ray Spectrometer (EDX) operated at 10 kV. The surface roughness of the coatings were measured by an atomic force microscope (AFM, Veeco DI-3100, Bruker, Beijing, China). The hardness and modulus of the as-deposited films were tested at five points for each sample with a nano-indenter using a Berkovich triangular pyramid indenter. The distance between each indentation was 50 μm. The Poisson’s ratio and Elastic modulus of the indenter tip were 0.07 and 1.141 × 10^6^ MPa, respectively. Micrographs of indentations were tested by a laser scanning confocal microscope (LSCM, OLS-4100, Olympus, Tokyo, Japan).

## 3. Results and Discussion

### 3.1. Phase Structures

#### 3.1.1. Phase Formation Mechanism Analysis

**Figure 2:**
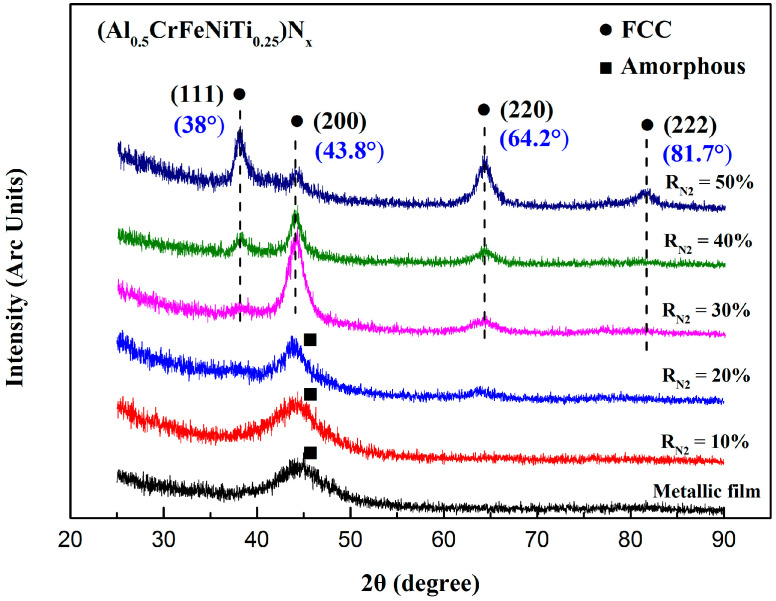
X-ray patterns of the (Al_0.5_CrFeNiTi_0.25_)N_x_ films deposited at different R_N2_.

**Figure 3:**
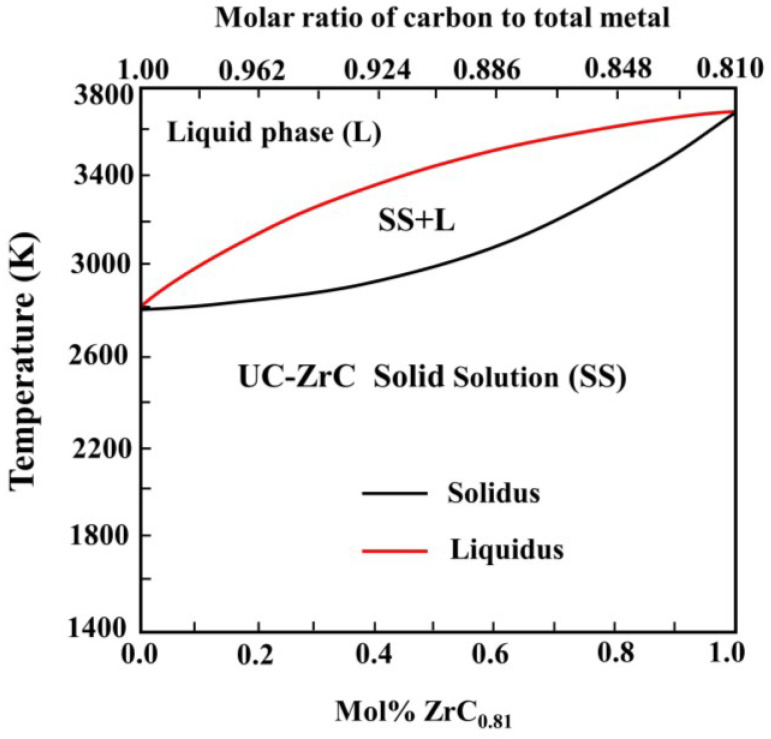
The UC-ZrC_0.81_ pseudo-binary phase diagram. Reproduced with the permission from Reference [34].

The XRD patterns of (Al_0.5_CrFeNiTi_0.25_)N_x_ films deposited at different R_N2_ are evaluated in Figure 2. An amorphous structure was observed with x = 0. As the N_2_ flow rate increased, the phase structures of (Al_0.5_CrFeNiTi_0.25_)N_x_ films showed a tendency to crystallize. Using XRD analysis, thin films displayed a simple FCC structure and the grains were nanoscale at about 20 nm. The bulk Al_0.5_CrFeNiTi_0.25_ alloys counterpart featured a BCC structure [27]. Due to the high-cooling rate, films were far from the equilibrium that could be achieved by bulk alloys, which led to the difference in structure even if they were the same system. Many similar results have been reported for other system film alloys [16,28,29,30,31]. While the metallic elements in this alloy system, which all had trends to form nitrides like TiN, CrN, AlN, and FeN, had results that showed only a simple FCC structure in an XRD pattern, rather than a various nitrides phase. It can be inferred that the solid solution occurred between nitrides in high-entropy thin films. Mutual dissolution between carbides and nitrides has been reported in many studies. Figure 3 shows the UC-ZrC_0.81_ pseudo-binary system that showed a miscibility relationship and the substitutional solid solution structure was formed with the carbides as solid solution units [32]. The nitrides also obtained similar results, such as Ti-Al-N, Cr-Al-N, Ti-Cr-N and Ti-Zr-N, Ti-Al-Si-N nitride films [33,34,35,36,37].

**Figure 4:**
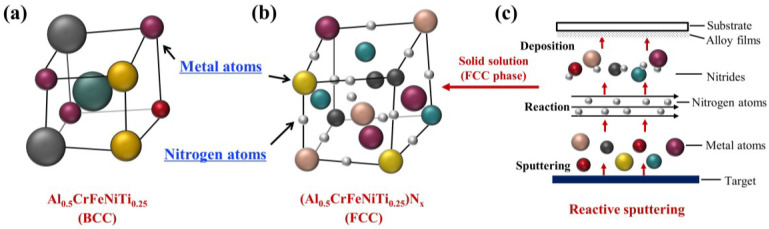
Schematic of lattice structure. (**a**) Al_0.5_CrFeNiTi_0.25_ bulk; (**b**) (Al_0.5_CrFeNiTi_0.25_)N_x_ films; and (**c**) schematic diagram of high-entropy thin film formation.

A previous study showed that FCC phases were found to have lower δ, whereas BCC phases showed higher δ [38]. Regarding (Al_0.5_CrFeNiTi_0.25_)N_x_ with x = 0, δ was approximately 6.4%, which was relatively large, and might form a BCC or ordered BCC structure. Metallic atoms solved each other and formed a BCC solid solution structure (Figure 4a). Accompanying the increase in nitrogen, nitrides formed in (Al_0.5_CrFeNiTi_0.25_)N_x_ and were similar in size and structure. The δ between nitrides was relatively small, thus an FCC structure might have formed. When R_N2_ was equal to 10% and 20%, a low concentration of nitrogen existed in the lattice. However, small amounts of nitrogen cannot facilitate lattice reconstruction. Thus, it still maintained an amorphous structure, however, with a higher nitrogen content, the formation of nitrides was promoted that efficiently improved the order of the lattice. The nitride components were becoming more similar in size and structure and solved each other easily, thus, an FCC (Al-Cr-Fe-Ni-Ti)N_x_ solid solution formed, which is shown in Figure 4b,c.

**Figure 5:**
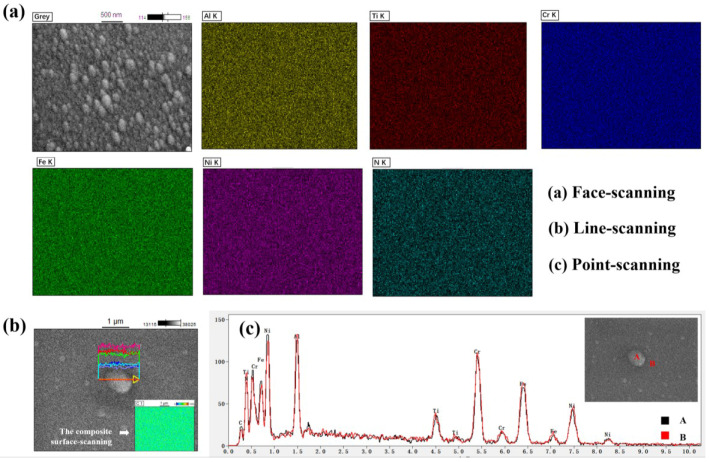
Energy dispersive X-ray spectroscopy of (Al_0.5_CrFeNiTi_0.25_)N_x_ high-entropy thin films deposited at R_N2_ = 50%: (**a**) face-scanning; (**b**) line-scanning; the insert picture is the composite surface scanning of this region; and (**c**) point-scanning.

Figure 5 displays the distribution of component elements of (Al_0.5_CrFeNiTi_0.25_)N_x_ thin films deposited at R_N2_ = 50% through EDX, including surface, and line and point scanning. As presented in each element map in Figure 5a, the distribution of component elements was uniform, which indicates a segregate-free characteristic. The results of line and point scanning also reflected the same element distribution characteristics. The internal picture in Figure 5b is the composite surface-scanning of this region and segregate-free characteristics can be observed. Viewing the comparative analysis of the energy spectrum of Point A and Point B in Figure 5c, the distribution of elements was stable and had a slight fluctuation. Moreover, the lattice constant of the FCC solid solution structure was calculated by the Prague formula. The FCC solid solution structure with a lattice constant about 4.093 Å was completely different with nitrides such as TiN, CrN, and so on, which also confirmed the occurrence of a solid solution.

#### 3.1.2. Solid Solution Formation Ability

Regarding high-entropy alloys, the solid-solution formation ability was estimated by the value of Ω and δ [39], which were defined as follows:

$$\Omega =\frac{T_{m}\Delta S_{mix}}{\left|\Delta H_{mix}\right|}$$

$$\delta =\sqrt{\sum_{i=1}^{n}c_{i}{\left(1-r_{i}/\bar{r}\right)}^{2}}$$

where *T_m_* was the melting temperature of the n-elements alloy, ∆*S_mix_* was the mixing-entropy of an n-element system, ∆*H_mix_* was the mixing-enthalpy of an n-element system, *c_i_* was the atomic percentage of the component, *r_i_* was the atomic radius, and $\bar{r}=\sum_{i=1}^{n}c_{i}r_{i}$ was the average atomic radius. Reviewing the previous study, it was concluded that the region where solid-solution structures were formed was in the range of 1.1 to 229.8 for Ω and 0.8% to 6.6% for δ. The radius of component elements in (Al_0.5_CrFeNiTi_0.25_)N_x_ are shown in Table 1, and the specific Ω and δ values of (Al_0.5_CrFeNiTi_0.25_)N_x_ at different N_2_ flow rates are shown in Table 2.

**Figure 6:**
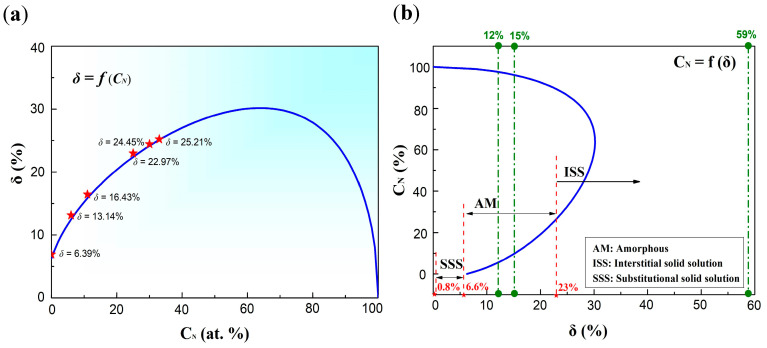
(**a**) Analytical curve of δ as a function of nitrogen content in (Al_0.5_CrFeNiTi_0.25_)N_x_ and (**b**) analytical curve of nitrogen content as a function of δ in (Al_0.5_CrFeNiTi_0.25_)N_x_ films (12% according to Inoue Principle, 15% and 59% according to the Hume–Rothery Rule).

Concerning (Al-Cr-Fe-Ni-Ti)N_x_, the δ was described as a function of nitrogen content, as shown in Figure 6a. The analytical curve was calculated based on the atomic ratio of the target, which was the theoretical value. While different deposition yields of component elements can cause fluctuations in the atomic ratio, the experimental values were well matched with theoretical values, as shown by the star points in Figure 6a. To clearly distinguish different phase regions of film alloys in this study, the nitrogen content was also described as a function of δ in Figure 6b. Regarding Al_0.5_CrFeNiTi_0.25_, δ was about 6.4% located in the SSS zone in Figure 6b, and the BCC solid solution phase structure was stable. When increasing the nitrogen content, the FCC structure formed in the (Al_0.5_CrFeNiTi_0.25_)N_x_ system. The calculated value of δ was about 23% for that FCC structure. When the nitrogen content was low, the amorphous structure was stable and located in the middle region.

### 3.2. Deposition Rates

**Figure 7:**
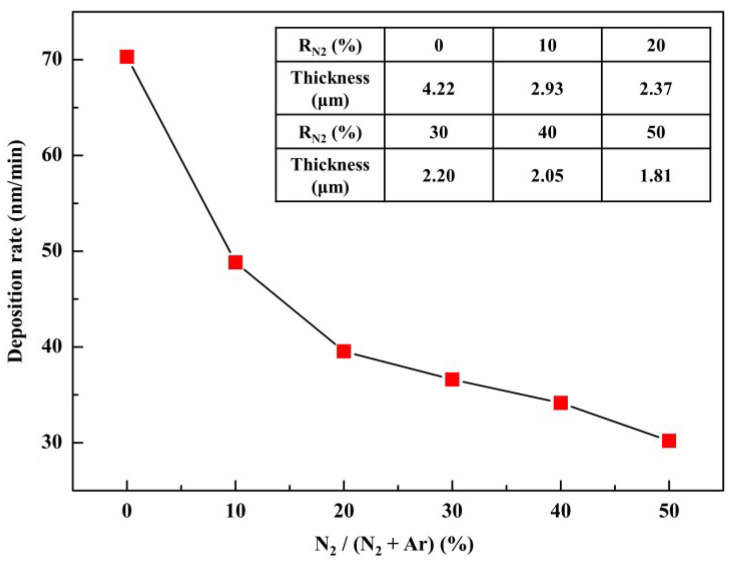
Deposition rates of (Al_0.5_CrFeNiTi_0.25_)N_x_ films at different R_N2_.

Figure 7 presents the deposition rate of (Al_0.5_CrFeNiTi_0.25_)N_x_ as a function of R_N2_ and the exact value of thickness is shown in the internal table. Following the addition of nitrogen, the deposition rate of films was significantly reduced by about 30%. During the deposition, the total flow rate of Ar + N_2_ was maintained at 30 sccm. Thus, the density of argon decreased gradually with the increase of N_2_ flow rate and resulted in a lower efficiency of argon-ion bombardment of the target. Under higher R_N2_, the deposition rate continued to slow and maintain a lower sputtering yield. Added to the effect caused by low argon gas density, the ceramicization of the target surface was also an important factor. This was mainly due to the N-containing layer that formed on the target surface, which reduced the conductivity of the target and resulted in a low sputtering yield. This phenomena was “target poisoning” [40,41].

### 3.3. Surface Morphologies

**Figure 8:**
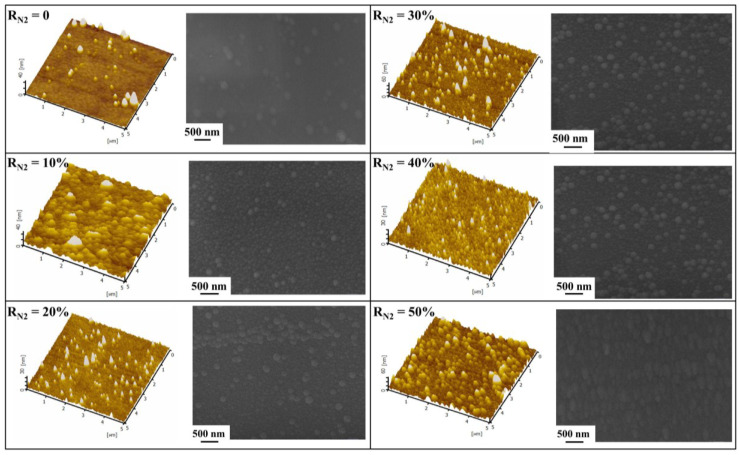
AFM images and SEM micrographs of (Al_0.5_CrFeNiTi_0.25_)N_x_ films at different R_N2_.

The AFM images and SEM micrographs of the (Al_0.5_CrFeNiTi_0.25_)N_x_ films at different R_N2_ are shown in Figure 8. Initially, a very dense and smooth surface with low surface roughness was obtained. Looking at SEM micrographs, it was observed that a small amount of nanostructure precipitation occurred as the R_N2_ increased to 10%. When the N_2_ flow rate increased, the number and particle size of the nanostructures were increased significantly. Viewing the AFM images, many needle nanostructures were also observed in the high-entropy thin films. Additionally, the roughness of films was measured by AFM. The value of R_a_ gradually became larger and the exact value of surface roughness at different R_N2_ is shown in Table 2. The heights of the needle structure in all the HEFs were less than 60 nm, which was consistent with the results since the as-deposited HEFs were smooth and homogeneous, as shown in SEM micrographs.

The surface nanostructures displayed by AFM images were different from each other and the size of the needle structures became larger with the increase of R_N2_. These nano-scaled needle structures were related to the structure zone models, which had a great impact on the microstructural evolution of thin film. Initially, as R_N2_ was low, the growth of the HEFs effect by nitrogen ions was limited. The nucleation barrier was generally expected to be small, which contributed to the formation of randomly oriented islands for films deposited on the silicon substrates without substrate heating. Accompanying the increase of R_N2_, a strong driving force was obtained, which facilitated the surface atom diffusion and grain boundary motion. To achieve a stable state, the overall surface and interface energy were minimized through forming the new islands. Islands with lower energy consumed the others. Thus, the roughness of the HEFs increased gradually with the increase of R_N2_.

### 3.4. Mechanical Properties

**Figure 9:**
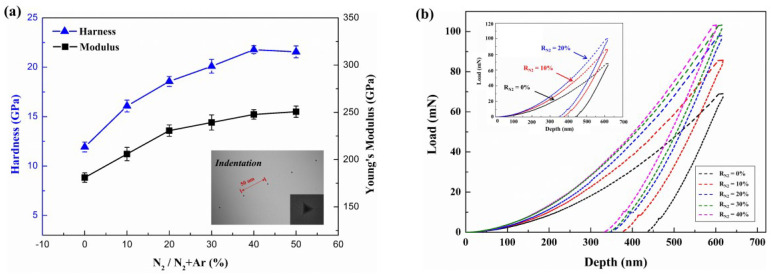
Mechanical properties of (Al_0.5_CrFeNiTi_0.25_)N_x_ films deposited at different R_N2_. (**a**) Hardness and modulus and (**b**) load-depth curve.

Figure 9a displays the hardness and Young’s modulus of (Al_0.5_CrFeNiTi_0.25_)N_x_ films as a function of the N_2_ flow rate. Through the increase of the nitrogen content, the hardness and Young’s modulus of the films showed a significant upward trend. The high-entropy thin films showed the highest hardness and Young’s modulus at 21.78 GPa and 253.8 GPa, respectively. Compared with the bulk Al_0.5_CrFeNiTi_0.25_ alloys (the value of hardness was about 6 GPa), the hardness of the (Al_0.5_CrFeNiTi_0.25_)N_x_ HEFs was improved significantly. Considering the load-depth curves of Figure 9b, the manifest and precise change of the hardness and Young’s modulus of the HEFs was obtained. When the probe depressed into the same depth, a larger force was required with the increase of the R_N2_. This was attributed to the fact that solid solution significantly increased the hardness. It is well known that solid solution strengthening increases the yield strength of the material by increasing the stress τ to move dislocations [42]:

$$\Delta \tau =Gb\varepsilon^{3/2}\sqrt{c}$$

where *c* was the concentration of the solute atoms, *G* was the shear modulus, *b* was the magnitude of the Burger’s vector, and *ε* is the lattice strain due to the solute. While increasing the N_2_ flow rates, the parameters *c* and *ε* were increased correspondingly, which resulted in a higher *τ* value. Thus, the effect of solid-solution strengthening was enhanced gradually with the increase of R_N2_. When the R_N2_ = 40% and 50%, the nitrogen content in films increased slowly. The difference in nitrogen content was only 2.66%, as shown in Table 2. The mechanical properties of the HEFs became stable.

## 4. Discussion

It is well known that the solid solutions can be divided into three types: Substitutional solid solutions; interstitial solid solutions; and vacancy solid solutions. Hume–Rothery rules are a set of basic rules that describe the formation of substitutional and interstitial solid solutions. Regarding a substitutional solid solution, the atomic radius of the solute and solvent atoms must differ by no more than 15%, while solute atoms should have a radius no larger than 59% of the radius of the solvent atoms for an interstitial solid solution [43]. However, the non-metallic elements, such as H, B, C, and N, having a very small atomic radius can form compounds with metal elements. When the ratio of a non-metal atom radius to a metal atom radius (R_x_/R_M_) is less than 0.59, the compounds have a simple structure such as M_4_X, M_2_X, MX, MX_2_, which is called an interstitial phase. When R_x_/R_M_ > 0.59, the compounds have a complex structure such as Fe_3_C, thus, there is a interstitial compound [44]. Regarding (Al_0.5_CrFeNiTi_0.25_)N_x_, the metallic elements all satisfy the formation rule for an interstitial phase and different nitrides with simple structures can be formed. The metallic elements will tend to be ionic and the ionic radius might well reflect the actual particle. Since most of the elements in this alloy system are transitional metals, their ionic radii are very close to each other. Nitride components are becoming more similar in size and structure and solve each other easily. Thus, the FCC structured phase is formed in (Al_0.5_CrFeNiTi_0.25_)N_x_.

**Figure 10:**
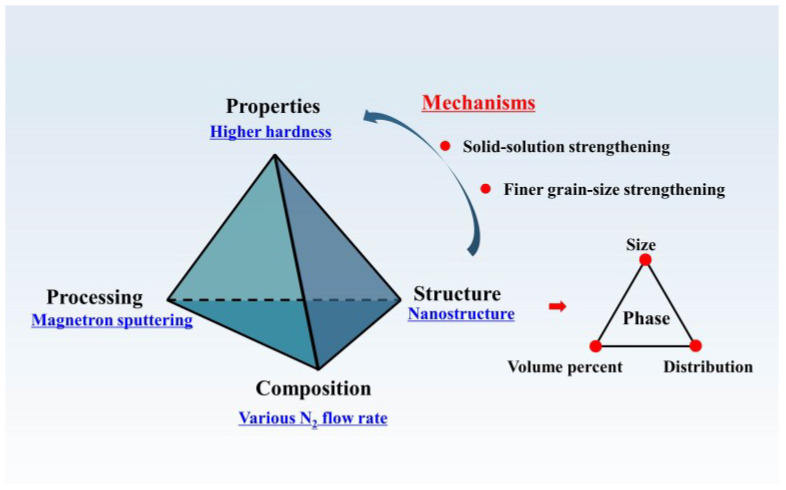
Relationship between composition, processing, properties, and the structures of high-entropy films.

The correlation between the composition, processing, microstructures, and the properties of high-entropy thin films can be summarized systematically and is shown in Figure 10. First, the films are prepared by a magnetron sputtering technique and different N_2_ flow rates are selected to adjust the composition as well as to change the microstructures. This finds that the nitrogen content in the thin films increases gradually with the increase of the nitrogen content and the structure of films transform from amorphous to FCC. Second, it is further found that the mechanical properties (hardness/Young’s modulus) of the high-entropy crystalline films are much better than the high-entropy amorphous films. All told, there is an increasing trend of hardness with higher nitrogen for the high-entropy thin films. Third, due to the difference in the preparation process, the phase structure and the properties of the films are different from the bulk samples, although they are in the same composition system. This study can help enrich the cognition between composition processing, microstructures, and the properties for high-entropy materials, especially for high-entropy thin films.

## 5. Conclusions

The (Al_0.5_CrFeNiTi_0.25_)N_x_ high-entropy thin films were deposited on silicon wafers through magnetron sputtering without substrate bias and heating. It was found that the phase structures of the HEFs transformed from an amorphous to an FCC structure with the increase in nitrogen content—the formation of a nitride solid solution was the main reason. Using the XRD analysis, the grain size was nanoscale at about 20 nm. The formation ability of the solid solution phase for the Al-Cr-Fe-Ni-Ti-N system film alloys was discussed regarding δ. When the value of δ was higher than 23%, the solid solution structures were stable in (Al_0.5_CrFeNiTi_0.25_)N_x_ thin films. The (Al_0.5_CrFeNiTi_0.25_)N_x_ HEFs deposited at R_N2_ = 40% and 50% yielded a maximum hardness and modulus of 21.78 GPa and 253.8 GPa, respectively, which is much higher than the as-cast Al_0.5_CrFeNiTi_0.25_ bulk alloys. The enhancement in hardness was mainly attributed to solid-solution strengthening and the lattice distortion. Additionally, smaller grain-size was a beneficial factor for increasing hardness.

## Figures and Tables

**Table 1:** The radius of component elelments in (Al_0.5_CrFeNiTi_0.25_)N_x_ films.

Element	N	Al	Ti	Cr	Fe	Ni
Radius (nm)	0.071	0.1434	0.1445	0.1249	0.1241	0.1246

**Table 2:** The nitrogen content, Ω value, δ value, and the roughness of films at different R_N2_.

R_N2_ (%)	0	10	20	30	40	50
Nitrogen Content (at %)	0	5.94	10.97	25.10	29.55	32.21
Ω	1.60	1.31	0.44	0.23	0.20	0.19
δ	6.39%	13.14%	16.43%	22.97%	24.45%	25.21%
R_a_ (nm)	1.375	1.574	3.780	4.542	4.490	5.574
